# The impact of tracheostomy delay in intensive care unit patients: a two-year retrospective cohort study

**DOI:** 10.1186/s40001-022-00753-5

**Published:** 2022-07-26

**Authors:** Anees Sindi

**Affiliations:** 1 Department of Anesthesia and Critical Care, Faculty of Medicine, King Abdulaziz University, Jeddah, Saudi Arabia; 2 King Abdulaziz University Hospital, King Abdulaziz University, Jeddah, Saudi Arabia

**Keywords:** Tracheostomy, Patient discharge, Intensive care units

## Abstract

**Aims:**

This study was undertaken to evaluate our tracheostomy service and identify reasons for any delays.

**Methods:**

A retrospective study in an academic tertiary-care hospital in Jeddah, Saudi Arabia. Inclusion criteria were any patients in ICU who required a surgical tracheostomy over a 2-year period (January 2014 to December 2015). The primary outcome was delayed tracheostomy referral and secondary outcomes included the number of days between referral and consultation, days between consultation and tracheostomy placement, and mortality rates.

**Results:**

Ninety-nine patients had a tracheostomy between January 2014 to December 2015 and could be analysed, mean age of 52.7 years, 44.5% females. The average duration from referral to tracheostomy was 5.12 days (SD 6.52). Eighteen patients (18.2%) had delayed tracheostomy (> 7 days from referral). The main reasons for the delay were the patient’s medical condition (50%, *n* = 9), followed by low haemoglobin (38.9%, *n* = 7). Administrative reasons were recorded in 5 cases only (28%); 2 due to operating room lack of time, 2 due to multidisciplinary issues, and 1 due to family refusal. Laboratory-confirmed low haemoglobin, a prescription of anti-platelets, or a prescription of anti-coagulation were not associated with a longer duration between referral and tracheostomy placement. An increase of 1 day in the time between referral and tracheostomy corresponded to an increase in delay in discharge from ICU of 1.24 days (95% CI 0.306 to 2.18).

**Conclusion:**

Although most delays related to the clinical condition of the patient, administrative and multidisciplinary factors also play a role. Early tracheostomy (less than 14 days) from intubation increases the survival rates of patients and improves their clinical outcomes. Further prospective evaluation is needed to confirm the impact of delay in performing surgical tracheostomy among ICU patients whose bedside percutaneous tracheostomy is contraindicated.

## Introduction

Prolonged stay in intensive care units (ICU) is associated with worse patient outcomes [1] as well as a logistic and financial burdens to hospitals [2]. The need for prolonged ventilation is one reason for patients requiring a longer stay in ICU, and this increases the risk of ventilator-associated pneumonia, acute respiratory distress syndrome, atelectasis, sepsis, and pulmonary oedema [3]. Even patients who do not require ventilation have increased mortality rates when the length of stay is prolonged [1]. Length of stay has been identified as an independent predictor for 1-year mortality, even when adjusting for age, comorbidity, red blood cell transfusion, low systolic blood pressure, pyrexia, and blood markers (total protein, platelet, and white cell count) [4].

Beyond optimising clinical care, a timely discharge from ICU is important in keeping costs down. Unfortunately, delayed discharge is quite common. In a study of 28,604 patients, nearly 70% had delayed discharge, accounting for 13% of total ICU bed-days [5]. Other studies report lower rates of delayed discharge, though even a 25% rate can lead to significant costs (199,268 Euros, equivalent to $240,000, per year) [6]. By far the most commonest reason for delayed discharge is the lack of appropriate ward beds, in some cases reaching 91% [6]. Despite that, other avoidable reasons should also be investigated and minimised.

Tracheostomy is frequently indicated in ICU patients either for long-term airway access or prolonged mechanical ventilation [7, 8]. Percutaneous tracheostomy is a commonly performed procedure that is usually performed by the ICU team; however, there are contraindications for this bedside procedure and when present, surgical tracheostomy is the preferred approach [8]. Surgical tracheostomy is routinely performed by the head and neck (H&N) team or by the ear, nose and throat (ENT) team in the operating theatre. This dependency on other teams outside of ICU is a factor that could lead to delays in discharge. Moreover, operating theatre booking is another critical factor.

Early tracheostomy placement has favourable patient outcomes. A study from 2006 showed that early tracheostomy in intensive care trauma patients improves resource utilisation, and delayed tracheostomy (> 14 days) was an independent predictor of prolonged ICU stay [9]. Similar findings are seen in systematic reviews and isolated studies, with most reporting statistically significant reductions in ICU length of stay and intubation-related complications when a tracheostomy is placed soon after intubation [9–11]. A study examining the effect of delayed tracheostomy (from the moment of decision to the procedure) on clinical outcomes found that shorter delays were also associated with successful weaning [12].

A tracheostomy should be performed as soon as possible after referral to the appropriate team in order to ensure a timely discharge and reduce complications. Therefore, our main objective of this study is to evaluate the timing of surgical tracheostomies after initial referral by the ICU team. As secondary objectives, we assessed and identified the factors affecting the delay and the impact of delays in service on ICU outcomes (length of stay in ICU and mortality rates). Finally, we evaluated the association between early tracheostomy (≤ 14 days from intubation) and ICU outcomes (length of stay and mortality). The information would help in delivering an efficient service to the benefit of our patients.

## Materials and methods

This was a retrospective cohort study in an academic tertiary-care hospital in Jeddah, Saudi Arabia. Inclusion criteria were any patients in ICU who required a surgical tracheostomy over a 2-year period (January 2014 to December 2015). Patients were identified from theatre logs, ICU logs, and referral logs to the department. Ethical approval was obtained from the institutional ethical committee.

Data obtained from the patients’ case notes included the date of ICU admission, date of referral to the surgical team, date of consultation by the surgical team, date of tracheostomy, and date of discharge from ICU. Patient factors included age, gender, haemoglobin (Hb) levels, prescription of anti-platelets or anti-coagulants, comorbidities, mortality, and cause of death. Reasons for delays in performing a tracheostomy were extracted from the patient records if available and categorised as: patient medical reasons (on anticoagulant or aspirin, medically unstable, low HB level), and administrative reasons (no available operative time, multidisciplinary issues, other).

For the main objective of evaluating service performance, the primary outcome was the duration (number of days) from referral for a tracheostomy to tracheostomy placement. We defined a delayed service when the time from referral to tracheostomy placement was longer than 7 calendar days, equivalent to 5 working days. Secondary outcomes included a number of days between referral and consultation, days between consultation and tracheostomy placement, and mortality rates. The study involved comparing the mortality rates involved deaths that occurred in ICU and days after discharge from ICU in terms of those who underwent early tracheostomy (less than 14 days after intubation) and late tracheostomy (more than 14 days after intubation). The secondary objectives of the study involved delayed service in ICU, outcomes related to the length of stay in ICU, and other miscellaneous factors related to the ICU.

Descriptive statistics were reported in frequencies, percentages, means and standard deviations (SD), ranges (minimum to maximum), interquartile ranges (IQR), and 95% confidence intervals (CI). Comparisons between groups were conducted with Chi-square tests and non-parametric tests (Mann–Whitney U). Statistical test was conducted to find any correlations between service performance indicators and ICU length of stay, and length between ICU admission and death occurring in ICU or within 30 days of discharge. Analyses were conducted with the Jamovi (version 1.6.9.0) statistical software, are two-tailed with an alpha level of 0.05, and reported with 95% confidence intervals (CI). As per the exclusion criteria, cases wherein critical components of the data were missing, were excluded from the study. It led to the strengthening of the study and showcasing the critical component of the study involved.

## Results

During the period January 2014 to December 2015, 107 surgical tracheostomies were performed. A total of 8 patients were excluded from any analysis because of missing referral dates or tracheostomy dates.

The characteristics of the 99 patients who had a tracheostomy during the study period are shown in Table 1. The average age of all cases was 52.7 years. There were 15 paediatric cases (< 18 years old), with a mean age of 4.5 years, while the mean age of adults was 61.3 years. There were 47 females (47.5%). The average haemoglobin was 9.74 gm/dL (SD = 2.13), median = 9.10 (range 7.0 to 17.4). Sixty-two patients (62.6%) had a haemoglobin of < 10 gm/dL, and 44 (44.4%) were on anticoagulation (warfarin or heparin) or antiplatelet therapy (aspirin) (Table 1).

**Table 1:** Characteristics of patients who had a tracheostomy during the study period

Characteristic	Value (*n* = 99)
Age, years—median (SD)	52.7 (26.1)
Adults (≥ 18)—*n* (%)	84 (84.8)
Male sex—*n* (%)	52 (55.5)
Haemoglobin, gm/dl	
Mean (SD)	9.74 (2.13)
Median (IQR)	9.10 (8.20–10.3)
< 10 gm/dL—*n* (%)	62 (62.6)
On anticoagulation—*n* (%)	39 (39.4)
On anti-platelets—*n* (%)	13 (13.1)
Had ventilation weaning—*n* (%)	45 (45.9)
Referral to tracheostomy, days	
Mean (SD)	5.12 (6.52)
Median (IQR)	3 (1–6)
> 7 days—*n* (%)	18 (18.2)
Referral to consultation, days	
Mean (SD)	0.5 (1.16)
Median (IQR)	0 (0–1)
Consultation to tracheostomy, days	
Mean (SD)	4.66 (6.47)
Median (IQR)	2 (1–5.75)
Overall mortality—*n* (%)	55 (55.6)
30-day mortality—*n* (%)	24 (24.2)

*SD* standard deviation, *IQR* interquartile range

### Service performance

The average duration between referral for tracheostomy and tracheostomy placement was 5.1 days (SD = 6.52), with a median of 3 days (IQR 1 to 6, range 0 to 35). Overall, in 18 cases (18.2%) the time between referral and tracheostomy placement was longer than a week (> 7 days), 9 (9.1%) of which had their tracheostomy 14 days or longer after referral. The duration between referral to consultation had a median of 0 days (range 0 to 9), and between consultation and tracheostomy a median of 2 days (range 0 to 35). The overall mortality rate was 55.6%, with half of the deaths occurring within 30 days after tracheostomy placement (Table 2).

**Table 2:** Service performance based on patient characteristics

	n	Referral to tracheostomy (days)		Referral to consultation (days)		Consultation to tracheostomy (days)	
		Median (IQR)	*P* value*	Median (IQR)	*P* value*	Median (IQR)	*P* value*
Haemoglobin			0.089		0.808		0.107
< 10 gm/dL	62	3 (1.25–7)		0 (0–1)		2 (1–6)	
≥ 10 gm/dL	37	2 (1–6)		0 (0–1)		1.5 (1–4.25)	
On anti-platelets			0.604		0.561		0.349
Yes	13	4 (2–6)		0 (0–0)		3 (2–5)	
No	86	3 (1–6)		0 (0–1)		2 (1–6)	
On anticoagulation			0.291		0.996		0.347
Yes	39	4 (2–6)		0 (0–1)		2 (1–5.5)	
No	60	2 (1–6)		0 (0–1)		2 (1–5.5)	

*IQR* interquartile range

^*^P values were calculated with the Mann–Whitney *U* test

### Reasons for delay

A reason for the delay in placing a tracheostomy after consultation was recorded in 15 cases out of the 18 who had a delayed tracheostomy (> 7 days). Six patients had multiple reasons documented. The commonest reason was the medical condition of the patient (50%, *n* = 9), followed by low haemoglobin (38.8%, *n* = 7). Administrative reasons were recorded in 5 cases only (27.8%); 2 due to issues with the operating room (lack of time), 2 due to multidisciplinary issues/coordination between the teams, and 3 due to others (1 no consent, 1 family refused initially). A laboratory low haemoglobin, a prescription of anti-platelets, or prescription of anti-coagulation were not associated with longer duration between referral and for tracheostomy placement (Table 3).

**Table 3:** Service performance of delayed tracheostomies based on documented reasons

	*n* (%)	Referral to tracheostomy (days)	Referral to consultation (days)	Consultation to tracheostomy (days)
		Median (IQR)	Median (IQR)	Median (IQR)
Medical	13 (72)	10 (9–19)	0 (0–0)	10 (9–19)
Low Hb	7 (39)	11 (10–16.5)	0 (0–1)	11 (9.5–16.5)
Medical other	9 (50)	10 (9–22)	0 (0–0)	9 (9–20)
Administrative	5 (28)	14 (14–21)	0 (0–0)	14 (14–21)
OR delay	2 (11)	14 (14–14)	0 (0–0)	14 (14–21)
Multidisciplinary	2 (11)	22 (15.5–28.5)	0 (0–0)	22 (15.5–28.5)
Admin other	3 (17)	21 (15–28)	0 (0–0)	21 (15–28)

IQR, interquartile range

### ICU outcomes

The average length of stay in ICU (admission to discharge) was 33.3 days (SD = 24.8), median 25 days (range 3 to 133), *n* = 50. Sixteen patients died while in ICU, 14 died ≤ 30 days after discharge from ICU, and another 17 afterwards. The average duration between admission and death in ICU was 46.5 days (SD = 21.2), median of 40 days (range 23 to 112), *n* = 16. Although mortality rates did not differ between patients who had fast service and those who had a delayed service, a fast service was associated with shorter ICU stays and longer survival (Table 4).

**Table 4:** Effect of service performance on ICU outcomes

	Fast service (≤ 7 days) *n* = 81	Delayed service (> 7 days) *n* = 18	*P* value*	All patients *n* = 99
Referral to tracheostomy, days			< 0.001	
Mean (SD)	2.72 (2.15)	15.9 (8.48)		5.12 (6.52)
Median (IQR)	2 (1–4)	12.5 (9–21)		3 (1–6)
ICU length of stay, days			0.004	
Mean (SD)	28.4 (19.5)	55.4 (34.6)		33.3 (24.8)
Median (IQR)	23 (17–38)	44 (32–63)		25 (18–39.8)
Admission to death, days†			0.072	
Mean (SD)	44.4 (32.5)	67.4 (38.3)		49.6 (34.6)
Median (IQR)	38 (27.3–53)	48 (46–80.5)		40 (29–55)
Overall mortality—*n* (%)	47 (58.0)	8 (44.4)	0.294	55 (55.6)
ICU mortality†—*n* (%)	23 (28.4)	7 (38.9)	0.381	30 (30.3)

*SD* standard deviation, *IQR* interquartile range

^*^P values were calculated with the Mann–Whitney U test or Chi-square test

^†^Death occurring in ICU or ≤ 30 days after discharge

In order to predict ICU length of stay in terms of tracheostomy referral and placement, a statistical test including correlation and regression analysis were implemented that evaluated the median (IQR) and compared patients who received tracheostomy (early—less than 14 days after intubation) and (delayed—14 days after intubation). The delay in service explained some of the amount of the variance in length of stay, *F *(1, 48) = 7.12, *p* = 0.010, *R*^2^ = 0.129. An increase of 1 day in the time between referral and tracheostomy corresponded, on average, to an increase in delay in discharge of 1.24 days (95% CI 0.306 to 2.18).

### Early versus late tracheostomy

The exact date of intubation was known in 82 patients A Mann–Whitney U test showed that the length of stay (admission to discharge) was shorter in patients who had early tracheostomy (intubation to tracheostomy ≤ 14 days) (mean = 17.5, SD = 9.74, median = 17, range 3 to 40, *n* = 21) compared to those who had late tracheostomy (> 14 days) (mean = 44.8 days, SD = 26.2, median = 38, range 18 to 133, *n* = 29), *p* < 0.001. Also, patients who had an early tracheostomy had shorter waiting times between the referral and the tracheostomy placement (Table 5).

**Table 5:** Early versus late tracheostomy

	Early tracheostomy (≤ 14 days) *n* = 40	Late tracheostomy (> 14 days) *n* = 42	*P* value*	All patients *n* = 99
Referral to tracheostomy, days			0.008	
Mean (SD)	3.52 (4)	7.31 (8.12)		5.12 (6.52)
Median (IQR)	2 (1–5)	4 (2–9)		3 (1–6)
ICU length of stay, days			<0 .001	
Mean (SD)	17.5 (9.74)	44.8 (26.2)		33.3 (24.8)
Median (IQR)	17 (11–21)	38 (25–49)		25 (18–39.8)
Admission to death, days†			0.007	
Mean (SD)	35 (16.1)	65.1 (42.5)		49.6 (34.6)
Median (IQR)	30 (24–41)	49 (41.5–64.5)		40 (29–55)
Overall mortality—*n* (%)	23 (57.5)	29 (69.0)	0.278	55 (55.6)
ICU mortality†—*n* (%)	6 (15.0)	8 (19.5)	0.693	30 (30.3)

*SD* standard deviation, *IQR* interquartile range

^*^P values were calculated with the Mann–Whitney *U* test or Chi-square test

^†^Death occurring in ICU or ≤ 30 days after discharge

## Discussion

**Fig. 1:**
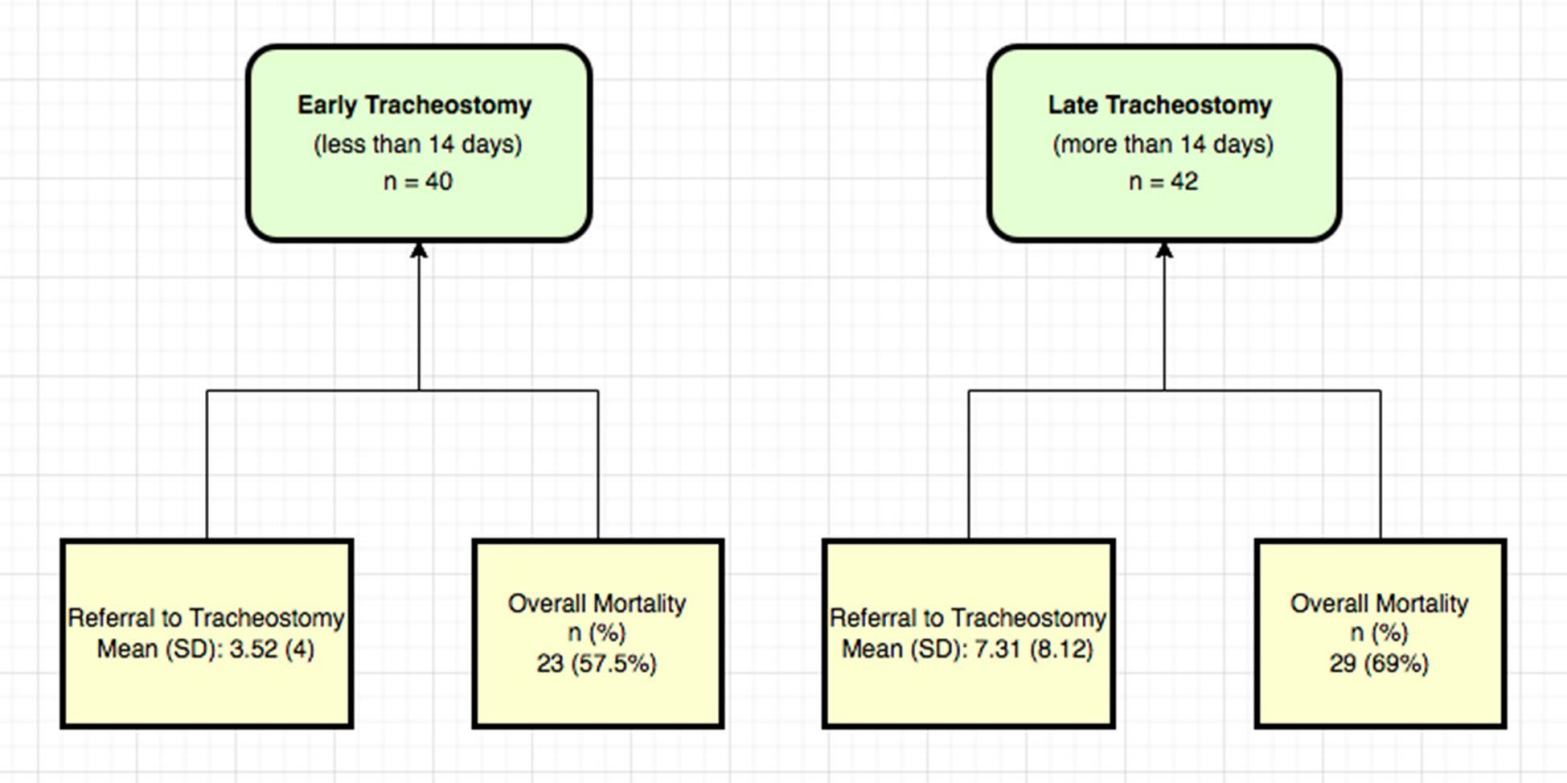
Flowchart of early versus late tracheostomy in terms of referral and overall mortality

This was a retrospective analysis of 2 years of data on surgical tracheostomy placement for patients in ICU. Patients were seen quickly after referral, and a tracheostomy was placed within 7 calendar days in 81.8% of the cases. Delays were primarily due to patient factors (medical conditions or low haemoglobin), and administrative reasons were recorded in only 5 (28%) of patients. These were due to coordination between different teams and in only 2 due to lack of theatre time (Fig. 1).

The delay between referral and tracheostomy placement is comparable to the only other study we could identify that reports similar data. In a retrospective cohort study of 134 tracheostomy patients, the average tracheostomy delay time (defined as the time from the physician’s suggestion of tracheostomy to the day of the procedure) was 4.4 days (SD = 5.7) [12], compared to 5.12 days (SD = 6.52) at our institution (assuming a referral was made the same day the ICU team suggested it). Tai et al. [12] reported that in their hospital in Taiwan, tracheostomy procedures are performed within 1 day of the patient's or family's decision and attribute cultural factors to any delays. However, 70% of their tracheostomies were percutaneous and this could account for the fast service. Similarly, possible regional and socioeconomic reasons were thought to be responsible for the considerable variation in the use of tracheostomy across 50 countries in a study of 2377 patients with acute respiratory distress syndrome [13]. Our results show that family factors were less relevant than other reasons, as in only one child the mother had delayed giving consent.

Our results indicate that for every extra day in the duration of delay referral and tracheostomy, discharge from ICU was delayed on average by 1.24 days, though this could be as little as a third of a day to two and a half days. The financial impact of these delays is significant both on a local level and a national level. Costs associated with a stay in ICU are high and account for a substantial portion of healthcare budgets [2]. In 2010, ICU costs in the United States (US) were estimated to be 13.2% of hospital costs [14]. Primarily, this is attributed to the usage of ventilation machines, expensive medication, and high salaries of ICU staff. For example, in the US the average salaries for members of the multi-professional ICU team per year range from $122,432 for acute care nurse practitioners, to over $3500,000 for intensivists [14]. Studies that estimate the cost per patient admission in ICUs have noted a significant variation. ICU costs were estimated to be $4,300/patient/day in 2010 in the US (a 61% increase since the 2000 cost of $2,669) [14], €3,980 ($4,740 equivalent) in 2017 in Norway [15], and €999 ($1,189 equivalent) for non-ventilated patients and €1,590 ($1,893 equivalent) for ventilated patients in Germany in 2020 [16].

Data from Saudi Arabia are lacking, with one of the largest ICUs in the country reporting costs of around 19,800 Saudi Riyals (SAR)/patient/day during 2017–2018 ($5,280 equivalent), with projected costs being reduced to < 18.000 SAR ($4,790 equivalent) after cost-cutting efforts [17]. The additional costs incurred due to delays in tracheostomy placement in our hospital, therefore, are averaged in the region of $6,550 per patient. During the 2 years of the study period, this equates to an estimated total of $350,280 per year. However, not all of these costs can be mitigated because the medical condition of a large number of patients prevented a timely surgical tracheostomy.

Although Tai et al. [12] do not report the effect of the delay from the decision to tracheostomy on other ICU outcomes such as length of stay or mortality rates, they identified that early tracheostomy within 14 days from intubation was associated with shorter mechanical ventilation duration (*p* = 0.001) and shorter ICU stay than late tracheostomy (mean = 16.6, SD = 8.5 vs mean 21, SD = 10.8 days, *p* = 0.009). In our results, early tracheostomy is also associated with shorter ICU length of stay compared to late tracheostomy (mean = 17.5, SD = 9.74 vs mean = 44.8, SD = 26.2, *p* < 0.001). The longer stay in ICU in our population is because of different populations and different ICU setup. Tai et al. examined patients requiring prolonged mechanical ventilation who are discharged to a specialised weaning respiratory care centre designed to improve the turnover rate of ICU [12]. The power analysis conducted (1-beta) was conducted for the study. The probability of a type 1 error in determining the differences between the 2 groups—early tracheostomy (less than 14 days after intubation) and late tracheostomy (more than 14 days after intubation), alpha is 0.05 and beta is the risk of type 2 error (false-negative rate). Beta is 0.2 and the power is 0.8. The sample size computed complies with the number of patients included in this retrospective cohort study.

The benefit of an early tracheostomy in ICU outcomes has been demonstrated in a variety of patient populations and settings. Alenazi et al. [18] found that early tracheostomy (≤ 12 days from intubation) was associated with shorter mechanical ventilation times and shorter ICU stay in patients with head injuries. This benefit appears to extend to even longer delays, as described by Mahafza et al. [10], when they compared patients who had a tracheostomy within 3 weeks of intubation, to those who had a tracheostomy later. A systematic review confirmed that early tracheostomy is associated with shorter ICU rates and patient outcomes, regardless of the cut-off point (7, 14, or 21 days) [19].

Although mortality rates were comparable between patients who had fast service (≤ 7 days) or not, and early tracheostomy or not, survival was shorter in those who either had a fast service or a tracheostomy within 14 days of intubation. Arabi et al. [9] reported that ICU mortality and hospital mortality were not different between the early (< 7 days post-intubation) and late tracheostomy. A review of the literature confirms that mortality rates are similar regardless of the cut-off period [11, 20], though we could not identify any literature pertaining to the delay between intubation and death. Our study excluded other confounding factors that could influence the mortality rates due to other medical causes by including deaths that occurred in the ICU and up to 30 days after discharge from the ICU.

## Limitations

The main limitations of this study are its retrospective nature and missing data. Because there is no formal guidance as to what constitutes a delay, it was left to the discretion of the clinician to document a reason. Not all delayed patients had a reason for a delay recorded in the notes, and this explains why there were discrepancies in some of the results. Our definition of delayed service (time from referral to tracheostomy > 7 days) can be considered generous, and more delayed cases could have been captured if we chose a shorter duration. However, the majority of patients who had a fast service had their operation within 4 days (median = 2, IQR 1 – 4). Gender does play a significant role for patients who suffered mortality due to delayed tracheostomy. However, our study primarily focussed on the role of early tracheostomy (less than 14 days after intubation) and late tracheostomy (more than 14 days after intubation). It could not be compared to the percutaneous tracheostomy. The sample size was evenly distributed with 52% males and 47.5% females. Finally, we did not account for the reason for intubation and the medical condition of the patient in the interpretation of the results. This information could provide further insight into why some patients had more delays than others, and why the true haemoglobin and prescription of anticoagulation/anti-platelet therapy did not appear to have an effect on the time between referral and tracheostomy. As per our retrospective cohort study conducted, the sample size was exposed to selection bias. However, we overcame the selection bias by focussing our study primarily on the clinical outcome of the patients in terms of early tracheostomy (less than 14 days after intubation) and late tracheostomy (more than 14 days after intubation). In this way, all the patients included in the study underwent tracheostomy and it reduced or minimised the selection bias and did not affect the outcome of the study.

## Conclusion

Delays in tracheostomy can incur significant costs to the hospital and can be associated with worse patient outcomes. Although most delays related to the clinical condition of the patient, administrative and multidisciplinary factors also may play a role. Further prospective evaluation is needed to confirm the impact of delay in performing surgical tracheostomy among ICU patients whose bedside percutaneous tracheostomy is contradicted. Early tracheostomy increases the survival rates of patients, especially less than 14 days of intubation. It can lead to the consumption of fewer ICU resources and improve patient outcomes.

## Declarations

### Availability of data and materials

The data pertaining to the study including OT logs, referral logs, and ICU data on percutaneous tracheostomies are available from the corresponding author upon request.
